# The impact of investing in private clinics and pharmacies on modern contraceptive uptake: an agent-based model of a segmented market

**DOI:** 10.1186/s12913-025-13334-z

**Published:** 2025-09-02

**Authors:** Mingxin Chen, Zhongzhe Pan, Y. Natalia Alfonso, Suzanne O. Bell, David Bishai

**Affiliations:** 1 Department of Population, Family and Reproductive Health, Bloomberg School of Public Health, Johns Hopkins University, Baltimore, MD USA; 2 Department of Health Policy, School of Public Health, Virginia Commonwealth University, Richmond, VA USA; 3 Department of International Health, Bloomberg School of Public Health, Johns Hopkins University, Baltimore, MD USA; 4 School of Public Health, The University of Hong Kong, Hong Kong , Hong Kong SAR China

**Keywords:** Family planning, Private sector, Public-private partnership, Agent-based model, Simulation, NetLogo, Contraceptive prevalence

## Abstract

**Background:**

The widespread availability of private-sector family planning services in low- and middle-income countries can be complementary to public-sector efforts to increase population coverage of modern contraceptive methods. The comparative advantages of a private-sector family planning program are more locations with shorter wait time. The disadvantages are higher prices and more variation in service quality. Our study quantifies how the balance of relative advantages and disadvantages might alter levels of contraceptive uptake in simulated urban and rural markets that present characteristics of markets throughout many low- and middle-income countries.

**Methods:**

We used agent-based simulation, a computational model that simulates the actions and interactions of artificial agents in a defined environment to understand how their collective behaviors affect outcomes of interest. Our agent-based model simulated one public vendor and one private vendor competing to serve a heterogeneous population of varied income and varied demand for higher service quality. We allocated new funding to one of the two policies: (1) invest in public-sector marketing and quality; or (2) invest in private-sector marketing, quality, and/or offer price support. Linear regression was applied to 6000 person-months of simulated outcomes per policy to compare modern contraceptive prevalence rates for each policy option.

**Results:**

The optimal policy differed for urban and rural market segments. Price reductions accompanying quality improvements in the private sector enhanced overall service utilization to the greatest extent. In the urban market, low-income women with a high preference for quality were the most responsive to price reductions from private vendors. In the rural market, high-income women with a high preference for quality were the most responsive to quality improvements while other population subgroups were responsive to price reductions in a similar manner.

**Conclusions:**

The factors that family planning policies should target to maximize their effect on modern contraceptive prevalence differ for markets characterized by different social contexts. Success of a policy will require policymakers’ attention to the relative preferences for low price, short wait time, short travel time, and quality among population subgroups. Our study results suggest that quality improvement in the private sector most benefit the urban populations, while price reductions are crucial for making family planning services more accessible to urban low-income populations and to rural populations across all income levels.

**Supplementary Information:**

The online version contains supplementary material available at 10.1186/s12913-025-13334-z.

## Background

The private sector is an important source of family planning (FP) services in low- and middle-income countries (LMICs), serving women of various socioeconomic backgrounds in both urban and rural areas [1–6]. A study in 11 low-income countries in Africa, Asia, and Latin America between 1987 and 1991 found that the private-sector share of the FP market ranged from 21.4 to 92.7% with a median of 35% [1]. More recent studies have continued to report similar results, with wide variation across countries [4–6]. As private FP services continue to play an important role in increasing access to FP services, it becomes critical to understand how harnessing the private sector or expanding public-private partnerships impacts contraceptive use. Substantial research focuses on public-sector strategies to improve population access to and use of modern contraceptives and reproductive health care services [7–9]. However, recent work indicates that public sector is unable to meet all current demand, especially for adolescent and young women, who often prioritize confidentiality when accessing short-term contraceptive methods. Interventions focusing exclusively on public-solutions for youth FP services may result in a coverage ceiling. Using DHS data from 33 sub-Saharan African countries in 2016, Radovich et al. found that younger women (aged 15–24) used fewer FP methods from the public sector than older women, and the private sector, especially commercial drug sellers, served as an important source of short-term methods for this population [10]. These preferences may stem from the private sector’s accessible locations, extended opening hours, quick service, and guaranteed confidentiality relative to public facilities [11]. In 2017, Bongaarts and Hardee used the Family Planning Program Impact score to assess the progress of government family planning programs in meeting FP demand in sub-Saharan Africa and showed that country progress varies significantly and remains particularly weak in Western and Central Africa [12].

Governmental family planning programs that seek to improve the delivery of modern contraceptives are operating in markets where there is already widespread use of the private sector for FP in LMICs [13, 14]. Thus, one alternative to scaling up public venues is to invest strategically in strengthening private-sector FP service providers so that they can meet demand while achieving social goals like serving the poor and offering high-quality contraceptive services. Research on strategies and policies to achieve this can help policymakers identify optimal policy options for different contexts.

In the private sector, key motivators for purchasing private FP services include convenience and perceived quality [15–17]. Utilization of private FP services typically increases by wealth in African and Asian LMICs [18]. However, despite the non-zero prices, private sector remains an important source of FP services among low-income women, primarily due to perceptions of higher quality of private FP services relative to public-sector alternatives [19]. Quality of FP services is multi-dimensional and includes the availability of respectful counseling about contraceptive choices and side effects, privacy, sensitivity to culture and context, range of services, method mix, appearance of the premises, technical knowledge and skill, and the condition of the products offered [20]. However, little is known about which dimensions of FP service quality drive demand the most [21, 22]. Lack of access to good quality and information on contraception is one main barrier that prevents women from seeking contraceptive options [23, 24]. Another barrier that determines the utilization of contraception is cost [25, 26]. In a broad sense, the definition of cost includes both the monetary prices of the FP services demanded, and the often non-monetized, opportunity cost of a woman’s efforts to obtain needed services, such as spending time and resources traveling to and waiting at a clinic.

Quality and price can be altered by both public-sector policymakers and private-sector providers. Left alone, the private sector will predictably not offer the optimal amount of access to FP services for the poor as private providers cannot sustainably lower their prices below their costs. Furthermore, because technical quality is not well assessed by private consumers and quality is costly to supply, private providers may not always invest heavily in maintaining quality service [16]. Data from an assessment of 346 facilities in Ethiopia and 1,099 facilities in Pakistan showed that a composite quality score based on Bruce’s quality domains was significantly lower in independent private clinics as compared to government clinics and franchised private clinics; franchised private clinics had slightly lower quality than government clinics but was the least accessible for the poor in Ethiopia [27]. Access to quality health services has been recognized as a human right, and it is the obligation of governments to assure quality in all facilities [28]. Improving service quality requires more capital, inventory, and training than are currently invested. Therefore, improving system performance in the private provision of FP requires investments to implement reform. Reforms might include regulating quality using sanctions and enforcing higher standards or investing in training and contact with private venues to improve their practices. There are also various approaches for policymakers to reduce the cost of FP services. The most direct method is to offer price support by subsidizing products and services. Other options include investing in additional human resources to reduce wait time and expanding the network of outlets by building new ones or partnering with existing ones to offer FP services.

One approach to determine which policies to employ would be to directly compare the impacts of the following strategies in a simulation: (1) Ignore the private sector and use all resources to expand public-sector provision; (2) Invest in making private-sector quality better (through training and/or measuring and regulating quality); (3) Invest in lowering prices in the private sector; (4) Spread information about private-sector services; (5) Combinations of the above. Each of these strategies would have advantages and disadvantages according to context. The number of possible combinations of strategies is extensive, and it would be difficult to empirically try out all the options in a community trial in a way that would lead to generalizable findings.

The goal of this paper is to rigorously define, examine, and model the role of information access, quality, and monetary prices in achieving better delivery of FP services in a setting where both private and public vendors are present. We present a detailed computer simulation of service supply and demand in a computer model using NetLogo. NetLogo is a simulation tool for multi-agent modeling of natural and social phenomena. It provides visual aids to present population behavioral changes in a complex system over time, and it is a powerful tool for organizing and utilizing findings from empirical studies to explore policy proposals and theoretical scenarios [29]. Examples of practical applications of NetLogo can be found in the fields of biology, engineering, sociology, economics, and epidemiology [30–32]. To the best of our knowledge, no existing studies have used NetLogo’s multi-agent simulation modeling feature to depict a community’s utilization of FP services. The paper will explore different FP policy options in the public sector versus the private sector, and the corresponding effects on different segments of the market. The paper will set out a typology of users and social contexts to shed light on which of the investment strategies generates the highest contraceptive coverage.

## Model setup

We simulated two FP vendors, one private and one public, to serve a simulated population of potential service users who inhabit a grid-like area. The private facilities charge a non-zero price that will be varied during policy experiments along with quality. The public facilities always charge a price of zero, but their quality can be varied across policy experiments.

**Fig. 1:**
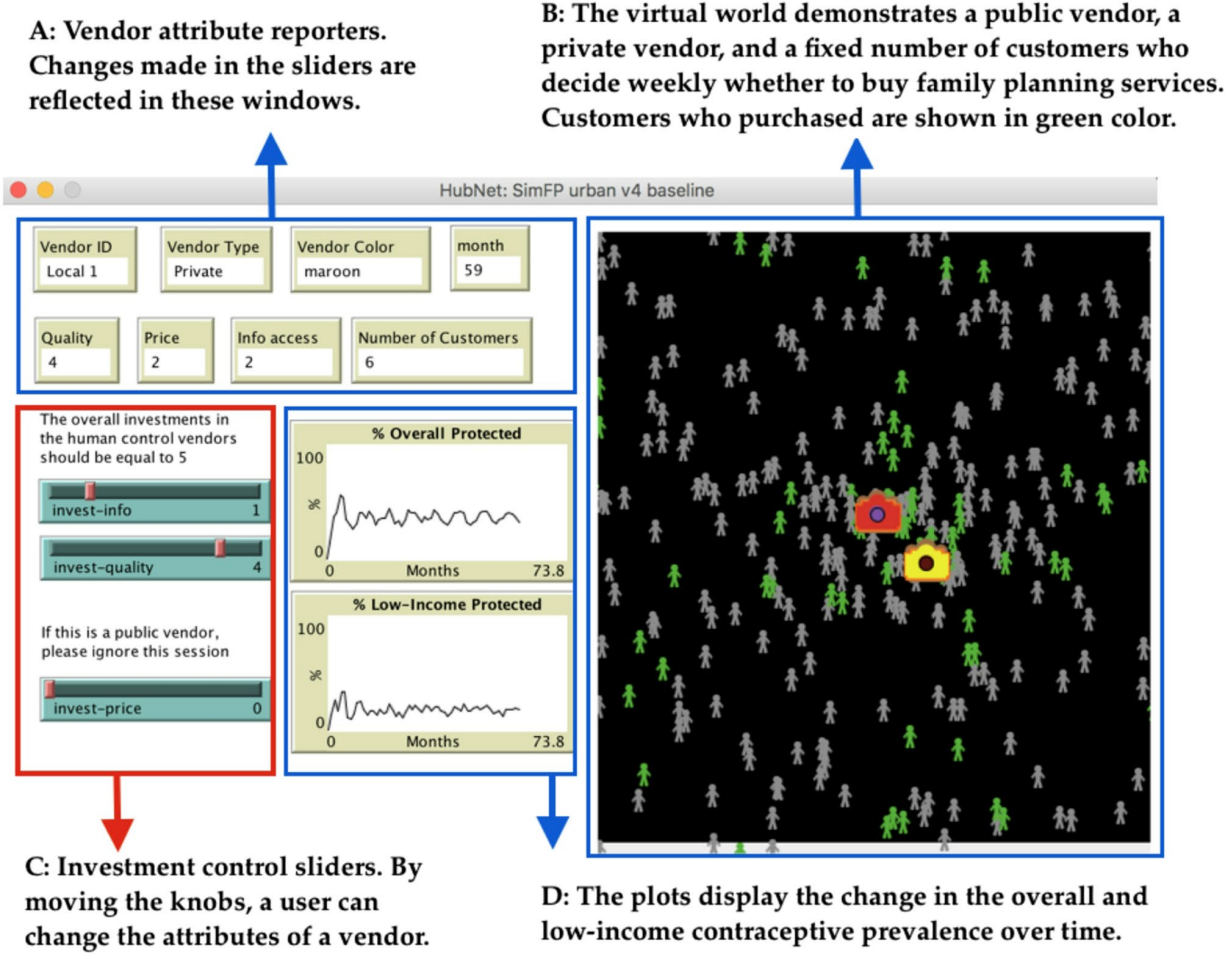
NetLogo’s vendor control interface for the urban model. The interface features investment control sliders for users to perform investment experiments

Urban and rural areas were simulated separately to assess how characteristics specific to each of these areas affect overall results. The urban model assumes travel time is negligible and features higher average wait time at the public vendor as compared to the private vendor. The urban public and private vendors are essentially co-located on the same spot on the grid so that travel time is the same. Therefore, the average cost of traveling to either vendor is the same and does not impact consumers’ decision-making. We made this modeling decision under the assumption that urban areas are more densely populated than rural areas, with health facilities being more concentrated and serving more people on average, given population density is one of the criteria that defines urban residence [33]. In contrast, we focus on travel time in the rural setting where individuals often face more distance- and transportation-related barriers to accessing health services [34]. The rural model features a travel cost that every consumer must pay per mile traveled. The mere existence of the second supply venue in the rural model reduces potential travel time for those consumers located far from the incumbent venue. Therefore, the rural public and private vendors are set to be distant from each other, located in different corners of the grid (Fig. 1). This makes half of the population closer to one vendor than the other. Wait time relative to travel time at rural vendors is negligible and therefore not incorporated into the rural model.

The simulated FP service users move around the grid to “shop” for FP services based on where they can get the best deal (to maximize their utility). Each service user is endowed with a unique income drawn from an income distribution and at any point each user is located at some geographic location on the grid and is either headed toward or away from the FP venues. To keep things simple, the model depicts only the members of a population who have a need for modern contraception. Each wants to avoid pregnancy, but each must balance this desire against what it might cost to obtain contraception in terms of money, wait time and travel time, as well as their preferences regarding the quality of care.

As noted, family planning quality has multiple dimensions such as method mix, adequate counseling, facilities, respectfulness, and technical skills. Models need boundaries and cannot depict all features of reality. We chose not to examine tradeoffs between the many dimensions of quality. In reality, various aspects of quality would be of different importance to various service users. Keeping a single aggregated concept of quality simplifies and allows policymakers to apply the results to whatever local dimensions of quality matter in their local context. Hence, quality’s multiple dimensions are not the focus of our model. The model collapses all possible dimensions of quality into one dimension that can vary from 0 to 5. Consumers are each endowed with their own intensity of preferences for quality drawn from a discrete distribution where half had a strong preference and the other half had a weak preference for quality.

Consumers must acquire information about FP quality directly during search efforts by visiting the premises of a provider. We also depict scenarios where the policymaker uses advertising to inform users about FP quality to spare consumers the effort of going to a provider who may or may not have sufficient quality services.

Each woman’s satisfaction level depends positively on earnings and negatively on the chance of unintended pregnancy. Each woman receives a weekly cash transfer equivalent to 1/52 of randomly assigned annual earnings, with half of the participants designated as high-income and the other half as low-income. They must decide whether to spend part of their earnings on FP each month. They can spend the remaining balance on other goods or services, but they cannot carry over unspent funds to future weeks, as the model does not include savings accounts.

The chance of unintended pregnancies is a function of service quality, which ultimately determines how satisfied consumers are with the FP services they receive [35]. User-side factors, such as method understanding and incorrect usage, are not considered since they largely reflect service quality. Consumers’ sensitivity to quality is randomly assigned so that half of the population have lower sensitivity, and the other half have higher sensitivity. If a woman has high sensitivity to FP quality, her demand will respond more to quality improvements at any given price than a woman with low sensitivity to FP quality.

We allow a single policymaker to try out the various policy options listed in Table 1 in either the public or private sector and track how they perform in terms of the resulting contraceptive prevalence rate. This policymaker has a fixed budget, which must be spent on some combination of quality improvements in public and private sectors, price reductions in the private sector, and/or providing users with better information about where to find higher quality services.

Table 1 outlines the hypothesized relationships between key access dimensions of FP services and consumer demand. We hypothesized that improvements in four specific access dimensions will independently increase demand: higher quality, lower prices, shorter wait time (urban setting) and shorter travel time (rural setting). These predictions identify potential targets for policy or program interventions. Because we endowed the women with idiosyncratic properties, we predict that low-income women will be more responsive to lower monetary prices; high-income women will be more responsive to shorter time spent waiting in an urban setting or traveling in a rural setting, as they are more able to trade money for time savings. Additionally, women who care more about quality will be more responsive to higher quality.

**Table 1:** Theoretically predicted direct effects of changes in access dimensions on demand for family planning services

Access Dimensions	Low-Income	High Income	High-Quality-Sensitivity
Higher Quality	↑	↑	↑↑↑
Lower Prices	↑↑↑	↑	↑
Shorter Wait Time (Urban)	↑	↑↑↑	↑
Shorter Travel Time (Rural)	↑	↑↑↑	↑

↑ Less responsive to the policy or social context change

↑↑↑ Responsive to the policy or social context change

Because these simple one-factor-at-a-time predictions are built into the model, the model is expected to display these patterns separately. What is not known a priori is what will happen when policymakers adopt multiple policies simultaneously. Furthermore, the private venues in urban areas are modeled to have a wait time advantage over public venues but also a built-in price and quality disadvantage, since private facilities may not consistently invest in maintaining quality due to the high associated costs [16, 27]. In a simulation, we can ask whether there is a scenario where the policymaker can achieve a net increase in contraceptive prevalence, by investing in either price supports or quality improvements in the private facilities.

## Methods of simulation

### Overview

We used NetLogo (version 6.0.1) as the platform for the model. The model’s agents are two FP vendors and a set of potential service users who all need contraception with different levels of preference for FP service quality. On the supply side, the public vendor is set to offer free services with slightly higher quality than the private vendor due to pre-existing endowments funded by government programs. The demand side features a virtual population of female FP consumers of reproductive age who have a need for FP, and can choose not to purchase, or to purchase at either a public vendor or a private vendor. Whether and where a consumer makes the purchase and the quantity they acquire depends on that individual’s budget and satisfaction level gained from lowering her risk of unintended pregnancy from the transaction, independent of other factors such as age, marital status, and number of children. This risk can be impacted by policy-related factors, service price and quality, as well as factors related to individuals and social context such as income, sensitivity to service quality, and cost of traveling or waiting.

Our model simulates different investment policies by distributing a fixed amount of public funds to either quality improvement at the public vendor, or quality improvement with or without price reductions at the private vendor (price is always 0 at the public vendor). Vendors that have improved their quality have the option to also pay for truthful advertising to increase information access about the quality of FP services at the facility. (Modeling false advertising would bring uninformative complexity to the model.) The model output shows how the policies influence modern contraceptive prevalence rates (mCPR) in various settings.

### Decision to purchase algorithm

Each week, consumers in the model examine whether they have at least one month of FP protection. If a consumer has zero months of remaining protection, the consumer determines whether they have sufficient funds to purchase services for that month. For consumers who can afford at least one unit (to cover one month) of FP services at either the public or the private vendor, they comparison shop and choose the vendor and quantity that maximizes their satisfaction level.

We assume consumers have access to perfect information about the money prices at the venues, but they are not fully informed about service quality until they visit a facility. This helps to depict the reality of information asymmetry about quality.

### Policy experiments

Figure 1 shows the model interface where policymakers can allocate a fixed amount of funds to improve information access, improve quality, and reduce price.

It is typical for simulations to have initial settings that are not near equilibrium. To accommodate this, we gave the model a 60-month burn-in period so that demand and supply would be at a stable equilibrium prior to the policy experiments. At month 61, the model re-allocated the public money to the private vendor in two different policy experiments. Distribution of the funds at the private vendor can be found in Tables 3 and 5.

### Sensitivity analysis

Drawing inference from a simulation is limited by the concern that any given model outcome is completely determined by a single set of assumed parameters. Sensitivity analyses allow one to assess how much model outcomes are being driven by parameter assumptions. When outcomes are seen across a range of parameter values, they are more likely to reflect features of the basic system being studied. Sensitivity tests changed fundamental parameters and were imposed at month 0 and lasted throughout the 120-month period. The content of each sensitivity analysis is explained in Table 2.

**Table 2:** Content of sensitivity analyses

Name	Content
Longer/Shorter Wait Time (Urban Only)	This experiment increased or decreased the wait time by 25% for all women
Longer/Shorter Travel Time (Rural Only)	This experiment increased or decreased the travel time by 25% for all women
FP-Resistant	This experiment reduced the willingness to purchase FP by 25% for all women regardless of venue
Reduced Effectiveness/Enhanced Effectiveness	This experiment made one unit of FP service 25% less or more productive in avoiding unintended pregnancy (UIP)
Consumption-Driven/Consumption-Indifferent	This experiment increased or decreased the utility gained from consuming one additional non-FP good by 25%; the former situation could make FP less desirable and the latter had the opposite effect
UIP-Driven/UIP-Indifferent	This experiment increased or decreased utility loss from the having one additional unintended pregnancy by 25%; the former situation could make FP more desirable and the latter had the opposite effect

### Policy models

Modern contraceptive prevalence rate (mCPR) was measured monthly for the first 60 months of burn-in and 60 months after the policy experiment in each iteration of each experiment. We conducted 100 iterations for each policy experiment with 100 women each time to generate data on the outcomes of 10,000 users. The model is stochastic because all the consumers were assigned a random starting point in their geospatial location relative to the FP venues. Each consumer also had a randomly drawn income and sensitivity to FP quality in each iteration. We sampled mCPR data from month 51–60 and 111–120 to ensure that an equilibrium was already reached before and after the investment policy shift for the analysis. Simple linear regression was applied to the dataset of 10,000 simulated women to analyze the association between mCPR and the public investment scenario (i.e., the policy implementation status).

The benchmark policy experiment addresses the question of whether switching funding from the public to the private sector could increase mCPR. In the benchmark we use 80% of the money drawn from the public sector to increase quality in the private sector and 20% of the money to spread information about the improved quality. The simple pre-post mCPR regression equation for the benchmark policy experiment is:

1 $$\:{mCPR}_{i}={\beta\:}_{0}+{\beta\:}_{1}*{Policy}_{i}+{\epsilon\:}_{i}$$

Where $\:{mCPR}_{i}$ is the mCPR in the ith iteration. $\:{Policy}_{i}$ is a dummy variable that takes the value 0 in pre-policy months and 1 in after-policy months. $\:{\beta\:}_{1}$ can be interpreted as the difference between pre- and post-policy mCPR. $\:{\epsilon\:}_{i}$ is the error term.

Tables 3, 4, 5 and 6 summarize the β1 results from each experiment. In each table, we regard the first-row policy as the benchmark and the second-row policy as the alternative. The remaining rows display the results of sensitivity analyses based on the benchmark policy. β1 results for the alternative policy and sensitivity analyses are bolded if their difference from the benchmark is statistically significant.

## Results

Table 3 shows the results of the urban model for overall and low-income consumers. The benchmark result in the first row indicates that reallocating investments away from public-sector quality to private-sector quality in the urban venue can raise mCPR by 8.6 percentage points in both overall and low-income populations. Compared to this benchmark, investing in both better quality and lower private-sector prices produces a much larger impact, particularly among low-income consumers.

**Table 3:** Urban model: policy experiments, social contexts, and corresponding simple linear regression (SLR) outcomes for the overall and low-income populations

Experiment Name	ΔOverall mCPR (%)	ΔLow-Income mCPR (%)	
POLICY EXPERIMENTS			
Benchmark:	8.64***	8.63***	
Invest 20% in perceived quality and 80% in true quality improvement at the private vendor	(0.10)	(0.11)	
Price Support:	39.68***	59.55***	
Invest 20% in perceived quality, 60% in true quality, and 20% in price support at the private vendor	(0.28)	(0.36)	
SENSITIVITY ANALYSES OF BENCHMARK POLICY			
Longer Wait Time:	9.32***	8.11***	
25% increase in the wait time	(0.10)	(0.09)	
Shorter Wait Time:	7.63***	9.30***	
25% decrease in the wait time	(0.10)	(0.11)	
FP-Resistant:	3.14***	0.21***	
25% decrease in the acceptance of FP services	(0.06)	(0.02)	
Reduced Effectiveness:	4.81***	3.19***	
25% decrease in the service effectiveness	(0.07)	(0.05)	
Enhanced Effectiveness:	−1.76***	0.00	
25% increase in the service effectiveness	(0.04)	(0.00)	
Consumption-Driven: 25% increase in the weight	7.40***	5.78***	
of disposable income in determining utility	(0.08)	(0.08)	
Consumption-Indifferent: 25% decrease in the	8.41***	10.13***	
weight of disposable income in determining utility	(0.14)	(0.13)	
UIP-Driven: 25% increase in the weight of	8.69***	9.89***	
UIP in determining utility	(0.12)	(0.13)	
UIP-Indifferent: 25% decrease in the weight of	7.19***	5.31***	
UIP in determining utility	(0.08)	(0.06)	

^a^ Standard errors in parentheses, *** p < 0.01, ** p < 0.05, * p < 0.1

^b^ Statistically significant improvements in the mCPR changes as compared to the benchmark model are shown in bold (p < 0.01)

The sensitivity analysis shows that the benchmark results are of similar overall magnitude despite alterations of 25% in the wait time, the marginal utility of consumption, or the marginal utility of UIP. However, the benchmark results appear sensitive to parameters that govern FP service effectiveness and the overall acceptance of FP. For example, when FP service is made 25% more effective in avoiding UIP across the board (regardless of quality), investments in private-sector quality no longer yield higher mCPR.

Table 4 reveals which sub-population is responsible for the response to quality improvements because it segments the population into four parts via a two-way tabulation of users across income and their sensitivity to quality. The first row of Table 4 shows that the impact of quality on mCPR is being driven by low-income women who are sensitive to service quality and by high-income women who are not sensitive to quality. The sensitivity analyses in Table 4 show continued sensitivity to the same parameters.

**Table 4:** Urban model: policy experiments, social contexts, and corresponding SLR outcomes for the four income/quality sensitivity subgroups

Experiment Name	ΔmCPR in Low-Income Low-Quality-Sensitivity	ΔmCPR in Low-Income High-Quality-Sensitivity	ΔmCPR in High-Income Low-Quality-Sensitivity	ΔmCPR in High-Income High-Quality-Sensitivity
POLICY EXPERIMENTS				
Benchmark	0.00	17.46***	16.00***	1.07***
	(0.00)	(0.20)	(0.22)	(0.27)
Price Support	69.21***	49.77***	41.18***	−1.24***
	(0.37)	(0.40)	(0.36)	(0.29)
SENSITIVITY ANALYSES OF BENCHMARK POLICY				
Longer Wait Time	0.00	16.31***	17.52***	3.39***
	(0.00)	(0.17)	(0.21)	(0.28)
Shorter Wait Time	0.34***	18.22***	12.16***	−0.37
	(0.03)	(0.22)	(0.23)	(0.27)
FP-Resistant	0.00	0.40***	3.02***	9.04***
	(0.00)	(0.04)	(0.14)	(0.24)
Reduced	0.00	6.39***	6.41***	6.41***
Effectiveness	(0.00)	(0.09)	(0.17)	(0.25)
Enhanced	0.00	0.00	−2.07***	−5.04***
Effectiveness	(0.00)	(0.00)	(0.11)	(0.20)
Consumption	0.00	11.55***	10.21***	7.72***
-Driven	(0.00)	(0.15)	(0.18)	(0.25)
Consumption	6.62***	13.71***	12.58***	0.66*
-Indifferent	(0.11)	(0.25)	(0.26)	(0.36)
UIP-Driven	5.75***	13.99***	14.39***	0.56*
	(0.08)	(0.23)	(0.26)	(0.32)
UIP-Indifferent	0.00	10.88***	8.55***	9.50***
	(0.00)	(0.12)	(0.16)	(0.24)

^a^ Standard errors in parentheses. *** p<0.01, ** p<0.05, * p<0.1

^b^ Statistically significant improvements in the mCPR changes as compared to the benchmark model are shown in bold (p<0.01)

In Table 5 the policy experiments are conducted in a rural population and show that the benchmark policy of reallocating funds from public-sector to private-sector quality lowers mCPR by 0.6 percentage points overall and lowers mCPR by 7.6 percentage points among low-income users specifically. However, adding price supports to quality investments does improve mCPR overall and among the poor. The sensitivity analyses in Table 5 show that the benchmark results are modified the most when travel time is reduced by 25%. In these models, the negative impact of the benchmark policy is reversed so that if travel time shortens, the investment in private-sector quality increases mCPR by 8.9 percentage points but reduces mCPR by 3.8 percentage points among low-income consumers.

**Table 5:** Rural model: policy experiments, social contexts, and corresponding SLR outcomes for the overall and low-income populations

Experiment Name	ΔOverall mCPR (%)	ΔLow-Income mCPR (%)	
POLICY EXPERIMENTS			
Benchmark: Partial Price Support	−0.63***	−7.66***	
Invest 20% in perceived quality, 60% in true quality improvement, and 20% in price support	(0.12)	(0.15)	
Full Price Support: 0 Price in Private Sector	31.93***	32.50***	
Invest 20% in perceived quality, 40% in true quality, and 40% in price support	(0.12)	(0.16)	
SENSITIVITY ANALYSES OF BENCHMARK POLICY			
Longer Travel Time:	−1.44***	−1.04***	
25% increase in the travel time	(0.11)	(0.15)	
Shorter Travel Time:	8.92***	−3.80***	
25% decrease in the travel time	(0.12)	(0.16)	
FP-Resistant:	−3.25***	**0.14**	
25% decrease in the acceptance of FP services	(0.11)	(0.16)	
Reduced Effectiveness:	−3.94***	−2.02***	
25% decrease in the service effectiveness	(0.12)	(0.16)	
Enhanced Effectiveness:	−2.75***	**0.08**	
25% increase in the service effectiveness	(0.11)	(0.15)	
Consumption-Driven: 25% increase in the weight	−1.53***	−3.22***	
of disposable income in determining utility	(0.11)	(0.15)	
Consumption-Indifferent: 25% decrease in the	3.69***	−9.83***	
weight of disposable income in determining utility	(0.11)	(0.15)	
UIP-Driven: 25% increase in the weight of	2.31***	−9.71***	
UIP in determining utility	(0.10)	(0.15)	
UIP-Indifferent: 25% decrease in the weight of	−1.94***	−2.27***	
UIP in determining utility	(0.12)	(0.16)	

^a^ Standard errors in parentheses. *** p<0.01, ** p<0.05, * p<0.1

^b^ Statistically significant improvements in the mCPR changes as compared to the benchmark model are shown in bold (p<0.01)

In Table 6, we see that the negative effects of the benchmark policy experiment are among low-income women and low-quality-sensitivity, high-income women. The benchmark policy increases mCPR among high-income, high-quality-sensitivity women. The effects of price support (row 2) are the same across all groups.

**Table 6:** Rural model: policy experiments, social contexts, and corresponding SLR outcomes for the four income/quality sensitivity subgroups

Experiment Name	ΔmCPR in Low-Income Low-Quality-Sensitivity	ΔmCPR in Low-Income High-Quality-Sensitivity	ΔmCPR in High-Income Low-Quality-Sensitivity	ΔmCPR in High-Income High-Quality-Sensitivity
POLICY EXPERIMENTS				
Benchmark	−2.67***	−12.65***	−4.95***	17.95***
	(0.22)	(0.21)	(0.24)	(0.24)
Full Price Support	31.21***	33.78***	32.38***	30.36***
	(0.24)	(0.24)	(0.26)	(0.23)
SENSITIVITY ANALYSES OF BENCHMARK POLICY				
Longer Travel Time	1.58***	−3.81***	−5.94***	2.30***
	(0.21)	(0.19)	(0.23)	(0.23)
Shorter Travel Time	−13.22***	5.44***	12.12***	31.24***
	(0.23)	(0.24)	(0.25)	(0.27)
FP-Resistant	2.21***	−1.91***	−5.86***	−7.40***
	(0.25)	(0.21)	(0.23)	(0.22)
Reduced	0.76***	−4.81***	−8.38***	−3.18***
Effectiveness	(0.20)	(0.23)	(0.24)	(0.25)
Enhanced	**−0.26**	**0.40**	−2.18***	−8.96***
Effectiveness	(0.22)	(0.26)	(0.25)	(0.20)
Consumption	1.00***	−7.50***	−6.90***	7.09***
-Driven	(0.22)	(0.22)	(0.24)	(0.23)
Consumption	−11.20***	−8.50***	7.04***	27.67***
-Indifferent	(0.22)	(0.23)	(0.22)	(0.23)
UIP-Driven	−9.89***	−9.44***	1.56***	27.16***
	(0.21)	(0.22)	(0.23)	(0.23)
UIP-Indifferent	1.84***	−6.41***	−6.74***	3.61***
	(0.24)	(0.22)	(0.23)	(0.25)

^a^ Standard errors in parentheses. *** p<0.01, ** p<0.05, * p<0.1

^b^ Statistically significant improvements in the mCPR changes as compared to the benchmark model are shown in bold (p<0.01)

## Discussion

Based on our a priori theory, we predicted that utilization of FP would be boosted by investments in private-sector quality (Table 2). The simulation results showed that these predictions were not uniform in all groups in all situations. In the urban market, results showed that women who were highly sensitive to quality responded to the quality increments in the private sector only if they were low-income, but not if they were high-income (Table 4). Conversely, in the rural market, women who were quality-sensitive responded to the private sector quality increments only if they were high-income, but not if they were low-income (Table 6).

The interaction between income and quality sensitivity was entirely unexpected and not pre-programmed into the model. Emergent results like this are typical of complex system models. One way to understand these surprising findings is to examine the parameter sensitivity results in the lower part of Tables 4 and 6. Here, we see that the high-income, high-quality-sensitivity women respond according to the prediction if their overall demand for FP is diminished by dialing down the parameters governing FP resistance, FP effectiveness, and the marginal utility of consumption and unintended pregnancy. One possible explanation is that a large proportion of urban high-income, high-quality-sensitivity women are already FP service users prior to the policy; they may switch vendors, but they are at ceiling levels of use before and after the policy. It is when these other reasons for using FP are dampened that they respond to quality increments as predicted. The results for rural women (Table 6) are analogous, with the responsiveness to quality becoming amplified when the parameters such as consumption indifference and UIP aversion are set to increase demand for FP.

Low-income, rural women who are set to be quality-responsive do not respond favorably to investments in private-sector quality regardless of parameter choice. One reason is that in the rural model, consumers spend down their budget simply by traveling to the venue. The low-income women who have traveled to a private venue for high quality have often spent so much on traveling that they cannot always afford the optimal level of FP services, regardless of how appealing those services are.

In Table 1, we predicted that lower prices would be especially appealing to low-income women. This prediction was not universally borne out. The simulation results on this in row 2 of Table 4 show that urban low-income women showed a higher demand response than their wealthier counterparts. Conversely, as seen in row 2 of Table 6, rural low-income women appear equally price responsive as high-income women.

We hypothesized that reduced wait time would be particularly appealing to urban women with higher wages. However, our findings in row 3–4 of Table 4 revealed the opposite: after investing in private-sector quality, contraceptive use among high-income urban women increases when wait time is 25% longer universally, and decreases when wait time is 25% shorter universally. One possible explanation is that when all facilities experience extended wait times, the greater absolute time savings (combined with better quality) at private facilities attract more high-income women, which leads to a slight increase in their contraceptive uptake. Furthermore, our model results in row 4 of Table 6 validated our prediction that when travel time decreases universally, high-income rural women respond more to the improvements in private facilities. This is consistent with the findings in earlier literature that private-sector midwifery clinics could improve rural women’s access to family planning services by reducing travel distance and transportation costs [36]. Lastly, row 3 of Table 6 shows investing in the private sector increases contraceptive use among low-income rural women when travel time increases. The reason may be complicated: for some women (especially those who live close to a private facility), the installation and quality improvements would be especially attractive. For others, the long travel time would cancel out the private-sector advantages, driving the women to public facilities for free FP services, despite their quality decline due to reallocation of funds from the public to the private sector.

## Limitations

Our study acknowledges several limitations. First, our model relies on significant simplifications that abstract away the complexity of real-world contexts. Specifically, it does not represent any specific geographic location or health systems, nor does it capture the heterogeneity and complexity of public and private health care sectors, or other factors that may influence where one accesses care.

Second, the model’s scope was intentionally narrowed to short-acting contraceptive methods to allow observation of multiple repeat purchases. This simultaneously constrained the comprehensiveness of the analysis and the generalizability of its results to long-acting contraceptive methods.

Third, the service users in the model had simplified economic lives. They were not endowed with more sophisticated economic behaviors such as saving money from their income, or increasing their monthly income.

Last but not the least, the model used a single quality index instead of a multidimensional metric that reflects various aspects of FP service quality. While necessary for advancing the model’s tractability, this approach does not yield insights into how investments in different quality dimensions impact access to and use of FP services, or allow comparing their relative effectiveness.

The primary objective of this model is to improve understanding of a family planning market by allowing experimentation and visualizing specific behavioral changes to stylized FP clinics. While acknowledging these limitations, we recognize that complicating the model to replicate more of the nuances of reality would make it harder to trace out policy counterfactuals. Future research might address these limitations by adapting the model to allow more granular representations of specific regions, populations, or health systems.

## Conclusions

This paper began with the insight that there would be potential tradeoffs between money price, quality, travel time, and wait time in the demand for FP. When we applied that insight to a simulated population that varied in their income and their responsiveness to FP quality, we found that simple predictions no longer held across the board. The response to better quality in urban private venues was greatest for low-income women, whereas it was greatest for high-income women in rural areas due to the high cost of travel in rural areas. There was a predicted advantage of private-sector vendors in offering shorter wait time in the urban setting. But this advantage was not uniformly attractive across income groups; higher income women were more likely to benefit from short waits. The simulation results emphasize that investments in service quality in the private sector are most likely to have the greatest impact on urban low-income women who have high sensitivity to quality and on some high-income women. In rural areas, quality investments alone had the most impact on high-income women with a high sensitivity to quality. Adding price subsidies to quality investments was an attractive policy in both urban and rural settings.

The main take-away lesson from both the theory and the simulation is that small variations among potential users matter in predicting the response to policy. Using public funds to improve quality or lower prices in private venues for family planning does not have the same impact everywhere. These investments should be used as remedies for particular health gaps in demand [37]. Policymaking requires attentiveness to the variations in the population being served.

## Supplementary Information

Supplementary Material 1.

## Data Availability

No datasets were generated or analysed during the current study.

## Abbreviations

FP: Family Planning
LMICs: Low- and Middle-Income Countries
mCPR: Modern Contraceptive Prevalence Rate
UIP: Unintended Pregnancy
