# Supplementary material for: The impact of investing in private clinics and pharmacies on modern contraceptive uptake: an agent-based model of a segmented market
Source: BMC Health Serv Res. 2025 Sep 2;25:1177. doi: 10.1186/s12913-025-13334-z (PMC12403456; doi:10.1186/s12913-025-13334-z)
Supplement: Supplementary file 1 — Supplementary Material 1. [file 12913_2025_13334_MOESM1_ESM.docx]

**Appendix: Difference-in-difference results for model comparison.**

To improve attribution of affect to modeled policies, a difference–in-difference (DID) analysis was used to address whether the mCPR change in an alternative policy or social context is statistically different from the mCPR change in the benchmark model. The DID regression equation is:

${mCPR}_{i}=\gamma_{0}+\gamma_{1}*{Policy}_{i}+\gamma_{2}*{Alternative}_{i}+\gamma_{3}*{Policy}_{i}*{Alternative}_{i}+\varepsilon_{i}$ (2)

A dummy variable, ${Alternative}_{i}$, is added, which takes a value 0 for the benchmark policy and 1 for an alternative policy or social context. $\gamma_{3}$ is the main parameter of interest, which can be interpreted as the difference in the change of mCRP before and after policy comparing simulated populations exposed to the benchmark and alternative model.

Tables S1-S4 show our difference-in-difference model results. The benchmark model serves as the reference model. Differences in post-intervention changes in mCPR (ΔmCPR) between alternative policy or social contexts and the benchmark model are reported.

| **Table S1. Urban setting: difference-in-difference (DID) outcomes for the overall and low-income populations in various policy experiments/social contexts compared with the benchmark** | | | |
| --- | --- | --- | --- |
| **Experiment Name** | **DID Overall mCPR**  **(%)** | **DID Low-Income mCPR**  **(%)** |  |
|  |  |  |  |
| **POLICY SCENARIOS** | | | |
| **Benchmark:** |  |  |  |
| Invest 20% in information access, and 80% in true quality at the private vendor | N/A | N/A |  |
| **Price Support:** | 31.04*** | 50.92*** |  |
| Invest 20% in information access, 60% in true quality, and 20% in price support at the private vendor | (0.30) | (0.38) |  |
| **SENSITIVITY ANALYSES OF HOW PARAMETERS CHANGE BENCHMARK POLICY** | | | |
| **Waiting-Averse:** | 0.68*** | -0.53*** |  |
| 25% increase in the effect of waiting | (0.14) | (0.14) |  |
| **Waiting-Tolerant:** | -1.01*** | 0.67*** |  |
| 25% decrease in the effect of waiting | (0.14) | (0.15) |  |
| **FP-Resistant:** | -5.50*** | -8.43*** |  |
| 25% decrease in the acceptance of FP services | (0.12) | (0.11) |  |
| **Reduced Effectiveness:** | -3.83*** | -5.44*** |  |
| 25% decrease in the service effectiveness | (0.12) | (0.16) |  |
| **Enhanced Effectiveness:** | -10.40*** | -8.63*** |  |
| 25% increase in the service effectiveness | (0.11) | (0.11) |  |
| **Consumption-Driven:** 25% increase in the weight | -1.24*** | -2.86*** |  |
| of disposable income in determining utility | (0.13) | (0.13) |  |
| **Consumption-Indifferent:** 25% decrease in the | -0.23 | 1.50*** |  |
| weight of disposable income in determining utility | (0.17) | (0.17) |  |
| **UIP-Driven:** 25% increase in the weight of | 0.053 | 1.26*** |  |
| UIP in determining utility | (0.16) | (0.16) |  |
| **UIP-Indifferent:** 25% decrease in the weight of | -1.45*** | -3.33*** |  |
| UIP in determining utility | (0.12) | (0.12) |  |

1. Standard errors in parentheses. *** p<0.01, ** p<0.05, * p<0.1.

| **Table S2. Urban setting: DID outcomes for the income/quality sensitivity subgroups in various policy experiments/social contexts compared with the benchmark** | | | | | |
| --- | --- | --- | --- | --- | --- |
| **Experiment Name** | **DID mCPR in**  **Low-Income**  **Low-Quality-Sensitivity** | **DID mCPR in**  **Low-Income**  **High-Quality-Sensitivity** | **DID mCPR in**  **High-Income**  **Low-Quality-Sensitivity** | **DID mCPR in**  **High-Income**  **High-Quality-Sensitivity** |  |
| **POLICY SCENARIOS** | | | | | |
| **Benchmark** | N/A | N/A | N/A | N/A |  |
|  |  |  |  |  |  |
| **Price Support** | 69.21*** | 32.31*** | 25.18*** | -2.32*** |  |
|  | (0.37) | (0.44) | (0.42) | (0.40) |  |
| **SENSITIVITY ANALYSES OF BENCHMARK POLICY** | | | | | |
| **Waiting-Averse** | 0.00 | -1.15*** | 1.52*** | 2.32*** |  |
|  | (0.00) | (0.26) | (0.31) | (0.39) |  |
| **Waiting-Tolerant** | 0.34*** | 0.76*** | -3.84*** | -1.44*** |  |
|  | (0.03) | (0.29) | (0.32) | (0.39) |  |
| **FP-Resistant** | 0.00 | -17.06*** | -12.98*** | 7.97*** |  |
|  | (0.00) | (0.20) | (0.26) | (0.36) |  |
| **Reduced** | 0.00 | -11.07*** | -9.59*** | 5.33*** |  |
| **Effectiveness** | (0.00) | (0.22) | (0.28) | (0.37) |  |
| **Enhanced** | 0.00 | -17.46*** | -18.07*** | -6.11*** |  |
| **Effectiveness** | (0.00) | (0.20) | (0.25) | (0.33) |  |
| **Consumption** | 0.00 | -5.91*** | -5.79*** | 6.65*** |  |
| **-Driven** | (0.00) | (0.25) | (0.28) | (0.37) |  |
| **Consumption** | 6.62*** | -3.75*** | -3.42*** | -0.41 |  |
| **-Indifferent** | (0.11) | (0.32) | (0.34) | (0.45) |  |
| **UIP-Driven** | 5.75*** | -3.47*** | -1.60*** | -0.51 |  |
|  | (0.08) | (0.30) | (0.34) | (0.42) |  |
| **UIP-Indifferent** | 0.00 | -6.58*** | -7.45*** | 8.43*** |  |
|  | (0.00) | (0.23) | (0.27) | (0.36) |  |

1. Standard errors in parentheses. *** p<0.01, ** p<0.05, * p<0.1.

| **Table S3. Rural setting: difference-in-difference (DID) outcomes for the overall and low-income populations in various policy experiments/social contexts compared with the benchmark** | | | |
| --- | --- | --- | --- |
| **Experiment Name** | **DID Overall mCPR**  **(%)** | **DID Low-Income mCPR**  **(%)** |  |
|  |  |  |  |
| **POLICY SCENARIOS** | | | |
| **Benchmark: Partial Price Support** |  |  |  |
| Invest 20% in information access, 60% in true quality, and 20% in price support | N/A | N/A |  |
| **Full Price Support: 0 Price in Private Sector** | 32.57*** | 40.16*** |  |
| Invest 20% in information access, 40% in true quality, and 40% in price support | (0.17) | (0.22) |  |
| **SENSITIVITY ANALYSES OF BENCHMARK POLICY** | | | |
| **High Travel Cost:** | -0.81*** | 6.63*** |  |
| 25% increase in the travel cost per fixed distance | (0.16) | (0.21) |  |
| **Low Travel Cost:** | 9.55*** | 3.86*** |  |
| 25% decrease in the travel cost per fixed distance | (0.17) | (0.22) |  |
| **FP-Resistant:** | -2.62*** | 7.80*** |  |
| 25% decrease in the acceptance of FP services | (0.17) | (0.23) |  |
| **Reduced Effectiveness:** | -3.31*** | 5.65*** |  |
| 25% decrease in the service effectiveness | (0.17) | (0.22) |  |
| **Enhanced Effectiveness:** | -2.12*** | 7.74*** |  |
| 25% increase in the service effectiveness | (0.17) | (0.21) |  |
| **Consumption-Driven:** 25% increase in the weight | -0.90*** | 4.44*** |  |
| of disposable income in determining utility | (0.17) | (0.21) |  |
| **Consumption-Indifferent:** 25% decrease in the | 4.32*** | -2.17*** |  |
| weight of disposable income in determining utility | (0.17) | (0.22) |  |
| **UIP-Driven:** 25% increase in the weight of | 2.95*** | -2.05*** |  |
| UIP in determining utility | (0.17) | (0.22) |  |
| **UIP-Indifferent:** 25% decrease in the weight of | -1.31*** | 5.39*** |  |
| UIP in determining utility | (0.17) | (0.22) |  |

1. Standard errors in parentheses. *** p<0.01, ** p<0.05, * p<0.1.

| **Table S4. Rural setting: DID outcomes for the income/quality sensitivity subgroups in various policy experiments/social contexts compared with the benchmark** | | | | | |
| --- | --- | --- | --- | --- | --- |
| **Experiment Name** | **DID mCPR in**  **Low-Income**  **Low-Quality-Sensitivity** | **DID mCPR in**  **Low-Income**  **High-Quality-Sensitivity** | **DID mCPR in**  **High-Income**  **Low-Quality-Sensitivity** | **DID mCPR in**  **High-Income**  **High-Quality-Sensitivity** |  |
| **POLICY SCENARIOS** | | | | | |
| **Benchmark** | N/A | N/A | N/A | N/A |  |
|  |  |  |  |  |  |
| **Full Price Support** | 33.88*** | 46.42*** | 37.32*** | 12.40*** |  |
|  | (0.32) | (0.32) | (0.35) | (0.32) |  |
| **SENSITIVITY ANALYSES OF BENCHMARK POLICY** | | | | | |
| **High Travel Cost** | 4.25*** | 8.84*** | -0.99*** | -15.66*** |  |
|  | (0.31) | (0.28) | (0.33) | (0.33) |  |
| **Low Travel Cost** | -10.55*** | 18.08*** | 17.07*** | 13.29*** |  |
|  | (0.32) | (0.32) | (0.34) | (0.36) |  |
| **FP-Resistant** | 4.88*** | 10.74*** | -0.91*** | -25.35*** |  |
|  | (0.33) | (0.30) | (0.33) | (0.33) |  |
| **Reduced** | 3.43*** | 7.84*** | -3.44*** | -21.13*** |  |
| **Effectiveness** | (0.30) | (0.32) | (0.34) | (0.35) |  |
| **Enhanced** | 2.42*** | 13.05*** | 2.76*** | -26.91*** |  |
| **Effectiveness** | (0.32) | (0.33) | (0.34) | (0.31) |  |
| **Consumption** | 3.67*** | 5.15*** | -1.95*** | -10.86*** |  |
| **-Driven** | (0.31) | (0.30) | (0.34) | (0.33) |  |
| **Consumption** | -8.53*** | 4.14*** | 11.99*** | 9.72*** |  |
| **-Indifferent** | (0.32) | (0.31) | (0.33) | (0.33) |  |
| **UIP-Driven** | -7.21*** | 3.21*** | 6.51*** | 9.21*** |  |
|  | (0.31) | (0.31) | (0.33) | (0.33) |  |
| **UIP-Indifferent** | 4.52*** | 6.23*** | -1.79*** | -14.34*** |  |
|  | (0.33) | (0.30) | (0.33) | (0.34) |  |

1. Standard errors in parentheses. *** p<0.01, ** p<0.05, * p<0.1.
